# The interactive association between sodium intake, alcohol consumption and hypertension among elderly in northern China: a cross-sectional study

**DOI:** 10.1186/s12877-021-02090-4

**Published:** 2021-02-23

**Authors:** Xi Nan, Haiwen Lu, Jing Wu, Mingming Xue, Yonggang Qian, Wenrui Wang, Xuemei Wang

**Affiliations:** 1 Department of Health Statistics, School of Public Health, Inner Mongolia Medical University, Jinshan Development District, Hohhot, 010110 China; 2 Department of Medical Imaging, Affiliated Hospital of Inner Mongolia Medical University, Inner Mongolia Medical University, Hohhot, 010050 China; 3 National Center for Chronic and Non-Communicable Disease Control and Prevention, Chinese Center for Disease Control and Prevention, Beijing, 100050 China; 4 School of Basic Medicine, Inner Mongolia Medical University, Hohhot, 010110 China; 5 Department of Chronic Disease Control and Prevention, Inner Mongolia Center for Disease Control and Prevention, Hohhot, 010031 China; 6 Inner Mongolia Center for Disease Control and Prevention, Hohhot, 010031 China

**Keywords:** Alcohol consumption, Dietary sodium, Hypertension

## Abstract

**Background:**

Hypertension is a worldwide public health problem. We sought to examine the interactive associations among sodium intake, alcohol consumption and hypertension among older adult residents of Inner Mongolia in northern China.

**Methods:**

This cross-sectional study used data from the National Survey for Nutrition and Adult Chronic Disease in Inner Mongolia. The prevalence of hypertension was age standardized by the direct method. Sodium intake and alcohol consumption were estimated using a weighing method and 24-h recalls on 3 consecutive days. Hypertension was either self-reported or field-measured. Participants were categorized into six subgroups according to combinations of sodium intake status and drinking level. Logistic regression was used to determine the interactive effect of sodium intake and drinking on hypertension.

**Results:**

Of the 820 older adults who participated in this study, 523 (63.80%, age-standardized rate = 62.33%) had been diagnosed with hypertension. The mean sodium intake was 4.88 g. Sodium intake and drinking excessively were both independently related to higher risk of hypertension. A formal test for a multiplicative interaction between sodium intake and drinking revealed a significant interaction (*p* = 0.042), and the multivariable-adjusted odds ratio (95% CI) for the interaction was 1.1 (1.0–1.3). After adjusting for confounders, compared with moderate sodium intake and no drinking group, the risk of hypertension was highest among those with both excessive sodium intake and excessive alcohol consumption, with an odds ratio of 3.6 (95% CI: 1.7–7.9).

**Conclusions:**

The study highlights the interactive effect of sodium intake and alcohol consumption on hypertension. Primary health care providers should pay special attention to older adults with hypertension—especially those with an unhealthy diet including both excessive sodium and excessive alcohol intake. These findings are applicable for older adults in Inner Mongolia and worldwide.

## Background

The burden of disease related to hypertension remains high. The Global Burden of Disease Study identified high systolic blood pressure as the largest contributor to global disability-adjusted life-years (DALYs) among Level 3 risks (accounting for 211.8 million global DALYs) in 2015 [1]. Notably, the bulk of hypertension cases are found among older adults, largely because of this group’s dramatically greater prevalence of the condition compared with that of younger people [2]. Hypertension is also more prevalent in low- and middle-income countries, where those are diagnosed with hypertension may not have the resources or the access to quality care necessary to successfully control their illness over the long term [3]. Nearly three-quarters of people who have been diagnosed with hypertension reside in low- and middle-income countries [4]. In China, population aging also represents a significant challenge for hypertension control.

Hypertension seriously affects quality of life for older adults [5]. However, hypertension can be prevented and controlled effectively by modifying lifestyle and dietary factors known to be associated with the condition [6, 7]—especially excessive sodium intake and heavy alcohol consumption. The global sodium intake survey found that the average sodium intake was 3–5 g/day in most countries [8]. However, in China, the problem of excessive sodium intake is serious. The fifth dietary survey conducted in China reported the mean sodium intake to be 14 g/day, which is double the World Health Organization’s recommended intake [9].

Additionally, heavy alcohol consumption has become increasingly recognised as a leading risk factor for the development of hypertension [10]. Worldwide, 32.5% of people were current drinkers in 2016, and the burden of disease caused by drinking increases with age, peaking in men aged 55–65 years [11]. A meta-analysis has shown that decreasing alcohol consumption, which is known to be an effective intervention, reduces the burden of disease caused by hypertension [12].

The prevalence of hypertension is higher in Inner Mongolia than in other parts of China because of this region’s relatively low rates of awareness, treatment and control, which lead to the earlier occurrence of hypertension in the life course [13]. Inner Mongolia’s less-developed economy and medical care system and the region’s residents’ lack of awareness regarding health care have resulted in poor public health conditions related to hypertension [14]. Therefore, hypertension has become a common chronic disease in people over 55 years old in Inner Mongolia, where the crude hypertension prevalence rate has been reported to be 54.10% [15].

High sodium intake and excessive drinking are prevalent in Inner Mongolia [16]. Both factors play an important role in the development of hypertension. Most evidence supports a positive association between sodium intake and blood pressure [17, 18], and the literatures on excessive alcohol consumption’s harmful effects on blood pressure level are also mostly consistent [19, 20]. However, little is known about the interactive effect of excessive sodium intake and drinking on hypertension in older adults. Therefore, with a view to expanding the present evidence base for the prevention of hypertension and developing future public health interventions in Inner Mongolia, we sought to use Chinese adult chronic disease and nutrition monitoring data to explore the association between the effects of sodium intake and alcohol consumption on hypertension in people over 55 years old in Inner Mongolia, particularly examining whether sodium intake and alcohol consumption have an additive or synergistic impact on hypertension and whether a positive or negative interaction is present.

## Methods

### Study design and participants

As part of the data collection for the National Survey for Nutrition and Adult Chronic Disease in Inner Mongolia, which was conducted across eight monitoring sites in Inner Mongolia, multi-stage stratified cluster sampling was used to ensure a cross-section of the study participants. The study design has been described previously [21]. The sample size was calculated using a 19.15% prevalence of hypertension as reported in the fifth health service survey in Inner Mongolia, error of 3%, a design effect of three and a non-response rate of 10%. In total, 820 individuals over 55 years old participated in the study. This survey was approved by the Ethical Committee of the National Institute for Nutrition and Food Safety of the Chinese Center for Disease Control and Prevention. Participation in this survey entailed no treatments or interventions that could impact the health of participants. All participants provided written informed consent before the start of the investigation.

### Data collection

#### Weighing method and 24-h recall surveys

A weighing method and 24-h recall surveys administered over three consecutive days were used to collect dietary data. Information about consumption of condiments, such as cooking oil, salt and monosodium glutamate, over three consecutive days was collected with a weighing method, using standardized weighing tools to weigh the amount of food to quantify consumption amount. The investigation lasted four days for each subject. On the first day, trained staff members visited the participant’s home and used a food scale to weigh and record all condiments, including the type of container used. On the second day, all condiments purchased and discarded were weighed and recorded, and the investigator repeated this procedure on each of the remaining days of the investigation. At the end of the survey, the remaining condiments were weighed, and the amount of condiments consumed by participants over three consecutive days was estimated. In the 24-h recalls, participants were asked to recall and describe all food and alcohol they had consumed over the same three consecutive days (two weekdays and one weekend day), except for condiments.

#### Questionnaire

Trained health facility personnel interviewed each participant face-to-face using a uniform questionnaire after obtaining informed consent in person. This questionnaire [21], which was developed for the National Survey for Nutrition and Adult Chronic Disease, included items on demographic characteristics (e.g., sex, age, education level), health status (e.g., hypertension, diabetes) and health-related behaviours (e.g., smoking, physical activity).

Questionnaires on physical activity covered three activity categories with twenty six items, including twenty items on physical activity and four items on rest and two items on sleep. These items asked the participants in what kind of activities they engaged, the frequency of activities per week and the total time spent on these activities per day. Physical activities were scored using the weighting procedure recommended by the International Physical Activity Guidelines for Americans [22], which calculates the total exercise metabolic equivalents (MET) of the three activity categories.

Participants’ height and weight were directly measured by trained and monitored workers, and blood sample was also collected.

### Measures

#### Sodium intake

The dietary sodium intake included the intake of condiments and sodium contained in food. The consumption of condiments was collected with a weighing method, and all food measure was assessed by the 24-h recalls. Firstly, the consumption of participants’ condiments and food were measured. Then, the sodium intake from each type of food was calculated using the China Food Ingredients Table (version II) [23]. Following the Chinese Nutrition Society, sodium intake was then categorized into two levels: sodium intake ≤2200 mg was defined as moderate, and sodium intake > 2200 mg was defined as excessive [23].

#### Alcohol consumption

A 24-h recall survey was used to estimate each individual’s alcohol consumption on three consecutive days. Beverage type (liquor with high alcohol content, liquor with low alcohol content, beer, yellow rice wine, rice wine or wine) and amount consumed were measured on three consecutive days. With one standard drinking unit equal to 10 g of alcohol, alcohol consumption was calculated according to the Manual of Chinese Chronic Disease and Nutrition Surveillance Survey [24]. Each participant’s average alcohol consumption was divided into three levels, following the 2016 Dietary Guidelines for Chinese Residents: never (0 g/day), moderate (men: ≤ 25 g/day, women: ≤ 15 g/day) and excessive (men: > 25 g/day, women: > 15 g/day) [24].

#### Definition of hypertension

The main outcome was hypertension, as indicated by meeting one of the following conditions. The first condition was self-reported hypertension—having a diagnosis of hypertension and currently receiving hypertension treatment [14]. The second condition was field-measured hypertension, assessed as the average of three blood pressure measurements carried out by trained investigators using an electronic blood pressure monitor (Model HBP1300, Omron, Japan) with a precision of 1 mmHg. A standardized protocol for blood pressure measurement was used, following the recommendations issued by the Chinese Working Group on Blood Pressure Measurement [25]. Measurements were taken when the participant was seated, after a rest period of at least 5 min. Blood pressure was measured three times, at 1-min intervals. Hypertension was defined as average systolic blood pressure ≥ 140 mmHg and/or average diastolic blood pressure ≥ 90 mmHg.

#### Definition of other variables

Ethnicity was categorized as Han, Mongolian or other minority. Marital status was categorized as single, married or other. Education level was categorized as primary school, junior high school, or high school and above. Smoking status was categorized as non-smoker (never having smoked), former smoker (previously smoked but quit) or current smoker (currently smoking). Total exercise MET was divided into tertiles, with physical activity categorized as low (total MET < 2988), medium (2988 ≤ total MET < 8400) or high (total MET ≥8400). Cut-offs for body mass index (BMI) were based on adjustments for the Chinese population issued by a working group on obesity in China [26]. BMI was categorized into three groups: < 23.9 kg/m^2^, 24.0–27.9 kg/m^2^ and ≥ 28.0 kg/m^2^.

#### Statistical analyses

The prevalence of hypertension was age standardized to the 2010 national demographic criteria in China using the direct method [27]. Chi-square tests were used to compare participants with and without hypertension in terms of demographic characteristics, sodium intake and drinking status. Additionally, jointly considering sodium intake status and drinking level, the participants were categorized into six subgroups: moderate sodium intake/no drinking, moderate sodium intake/moderate drinking, moderate sodium intake/excessive drinking, excessive sodium intake/no drinking, excessive sodium intake/moderate drinking and excessive sodium intake/excessive drinking.

Initially, we analysed the independent associations of sodium intake and drinking with hypertension by estimating odds ratios (OR) and 95% confidence intervals (CIs) using multivariable logistic models. A multiplicative interaction term between sodium intake and drinking was also included in the logistic regression models to test whether this interactive effect on hypertension was independent of sodium intake, drinking and other confounding factors. Then, a logistic regression model was used to compute the ORs for hypertension across the six subgroups by adjusting for important confounding factors to explore the main interaction effects of sodium intake and drinking on hypertension. We established three multivariate models: Model 1 was an unadjusted model. Model 2 adjusted for demographic variables including sex, ethnicity, educational level, marital status, BMI and family history of hypertension. Model 3 further adjusted for smoking, physical activity, diabetes and dyslipidaemia. Finally, the single effect of sodium intake and drinking on hypertension was compared.

The ‘Forward: LR’ method was used to select variables in the logistic regression. Statistical significance was set at *α* < 0.05. All statistical analyses were performed with SPSS, Version 19.0 (IBM Corp, Armonk, NY, USA).

## Results

### Characteristics of Inner Mongolia residents

Using the 2010 national demographic criteria for age standardization, of a total of 820 residents aged 55 years and older, 523 (63.80%, age-standardized rate = 62.33%) had been diagnosed with hypertension. Compared with residents without hypertension, participants with hypertension were more likely to be Mongolian and to have a high BMI, a family history of hypertension, lower physical activity, and diabetes or dyslipidaemia (Table 1). There were no differences in the other variables between participants with and without hypertension.

**Table 1:** Characteristics of Inner Mongolia study participants aged over 55 years

Variables	Total, n	Hypertension, %	***χ*** ^**2**^	***p***-value
**Sex**			0.097	0.756
Male	411	63.26		
Female	409	64.30		
**Ethnicity**			4.772	0.092
Han	728	62.91		
Mongolian	63	76.19		
Others	29	58.62		
**Education level**			3.106	0.212
Primary school	514	64.40		
Junior high school	172	66.86		
High school and above	134	57.46		
**Marital Status**			3.128	0.209
Single	6	33.33		
Married	743	63.39		
Others	67	68.66		
BMI^*^			23.368	< 0.001^^^
<23.9	309	55.34		
24 ~ 27.9	344	67.15		
≥ 28.0	154	77.27		
**Family History of Hypertension**			22.673	< 0.001^^^
No	556	58.27		
Yes	264	75.38		
**Smoking Status**			4.182	0.124
None-Smoking	466	65.24		
Former Smoking	75	70.67		
Current Smoking	279	57.74		
**Physical Activity**			12.112	0.002^&^
Low	326	70.86		
Medium	255	60.39		
High	239	57.74		
**Diabetes**			7.133	0.008^&^
No	711	62.03		
Yes	109	75.23		
**Dyslipidemia**			6.224	0.013^&^
No	447	59.96		
Yes	373	68.36		

### Associations among sodium intake, drinking and hypertension

The average salt consumption was 9.78 g/day, and the mean sodium intake among the study participants over 55 years old was 4.88 g/day. Participants with excessive sodium intake and who consumed excessive amounts of alcohol had the highest rates of hypertension (Table 2). Table 2 also presents the independent effects of sodium intake and drinking on hypertension risk. After adjustment for confounders, excessive sodium intake was independently related to the risk of hypertension (OR = 1.9, 95% CI: 1.2–2.8). The odds of hypertension were twice as high for participants with excessive drinking compared with non-drinkers (OR = 2.0, 95% CI: 1.1–3.5). However, the odds of hypertension were not significantly higher for those who reported moderate drinking than for the non-drinking group. A formal test for a multiplicative interaction between sodium intake and drinking revealed a significant interaction (*p* = 0.042), and the multivariable-adjusted OR (95% CI) for the interaction was 1.1 (1.0–1.3) (Table 2).

**Table 2:** Prevalence and odds ratios of hypertension for sodium intake and drinking

Variables	Total, n	Hypertension, %	***χ*** ^**2**^	***p***-value	Unadjusted		***p***-value	Adjusted^**a**^		***p***-value
					***OR***	95%CI		***OR***	95%CI	
**Sodium intake**			6.921	0.009^&^						
Moderately	112	52.68			1.0(Ref)			1.0(Ref)		
Excessively	708	65.54			1.7	(1.2, 2.6)	0.007^&^	1.9	(1.2, 2.8)	0.003^&^
**Drinking**			6.681	0.035^&^						
No drinking	600	63.33			1.0(Ref)			1.0(Ref)		
Moderately	147	59.18			0.8	(0.6, 1.2)	0.265	0.9	(0.6, 1.3)	0.495
Excessively	73	76.71			1.9	(1.1, 3.4)	0.027^&^	2.0	(1.1, 3.5)	0.022^&^
**Sodium Intake×Drinking**	–	–	–	–	1.1	(1.0, 1.3)	0.042	1.1	(1.0, 1.3)	0.032^&^

^**a**^Adjusted for age, sex, marital status, education level, smoking, physical activity, BMI(Body mass index), diabetes and dyslipidaemia; ^&^*p* < 0.05; *OR* odds ratio, *CI* confidence interval

### Sodium intake–drinking interaction and risk of hypertension

**Fig. 1:**
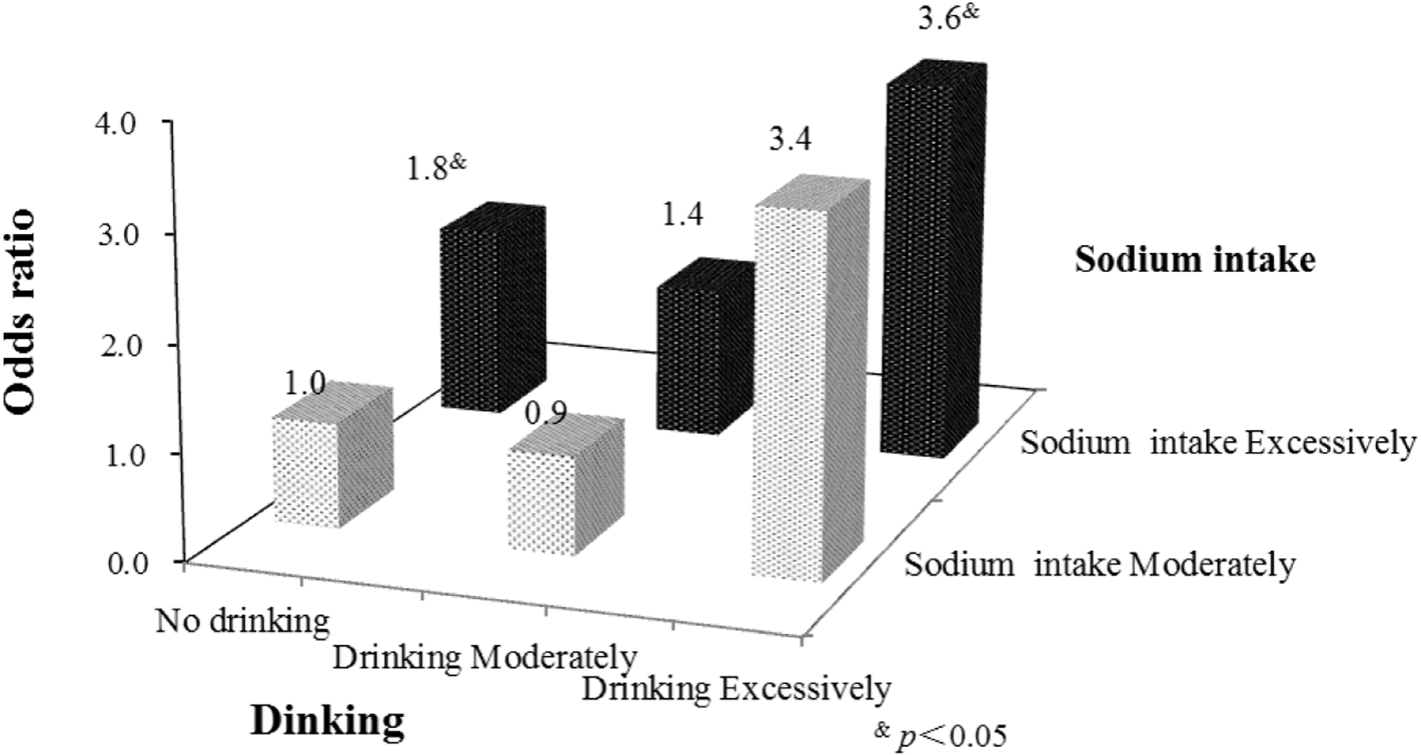
Interactive association of sodium intake and drinking with hypertension

When participants were categorized into six subgroups considering sodium intake and drinking jointly, the excessive sodium intake/excessive drinking group had the highest prevalence of hypertension, whereas non-drinkers with moderate sodium intake had the lowest prevalence of hypertension (*p* = 0.013) (Table 3). Table 3 shows the interactive effect of sodium intake and drinking on hypertension by comparing these six subgroups. After adjusting for demographic variables, the excessive sodium/non-drinking group and the excessive sodium/excessive drinking group displayed higher risks of hypertension compared with the moderate sodium/non-drinking group, with ORs of 1.9 (95% CI: 1.2–2.9) and 3.4 (95% CI: 1.7–7.1), respectively. After further adjustment for smoking, physical activity and disease-related variables, those with excessive sodium intake and excessive drinking had the highest risk of hypertension compared with those in the moderate sodium/non-drinking group, with an OR of 3.6 (95% CI: 1.7–7.9). The odds of hypertension were 1.8 times higher for participants in the excessive sodium/non-drinking group (adjusted OR = 1.8, 95% CI: 1.1–3.0) and 3.6 times higher for participants in the excessive sodium/excessive drinking group (adjusted OR = 3.6, 95% CI: 1.7–7.9) than for those in the moderate sodium/non-drinking group (Fig. 1).

**Table 3:** Logistic regression analysis of the interaction effect of sodium intake and drinking on hypertension

Variables	Total, n	Hypertension, %	***p***-value^**&**^	Model 1^**a**^	Model 2^**b**^	Model 3 ^**c**^
				OR(95%CI)	OR(95%CI)	OR(95%CI)
**Subgroups**			0.013			
Sodium moderately (No drinking)	89	50.56		1.0(Ref.)	1.0(Ref.)	1.0 (Ref.)
Sodium moderately (Moderate drinking)	13	53.85		1.1 (0.4, 3.7)	0.8 (0.2, 2.8)	0.9 (0.3, 3.3)
Sodium moderately (Excessive drinking)	10	70		2.3 (0.6, 9.9)	2.9 (0.6, 15.1)	3.4 (0.6, 17.7)
Sodium excessively (No drinking)	511	65.56		1.9 (1.2, 2.9)^&^	1.7 (1.1, 2.7)^&^	1.8 (1.1, 3.0)^&^
Sodium excessively (Moderate drinking)	134	59.7		1.4 (0.8, 2.5)	1.2 (0.7, 2.1)	1.4 (0.8, 2.6)
Sodium excessively (Excessive drinking)	63	77.78		3.4 (1.7, 7.1)^&^	3.3 (1.5, 7.0)^&^	3.6 (1.7, 7.9)^&^

^a^Unadjusted model; ^b^Adjusted for sex, ethnicity, education level, marital status, BMI(Body mass index) and family history of hypertension; ^**c**^Further adjusted for smoking, physical activity, diabetes and dyslipidaemia; ^&^*p* < 0.05; OR: odds ratio; CI: confidence interval

### Simple effect on hypertension at different levels of sodium intake and drinking

**Fig. 2:**
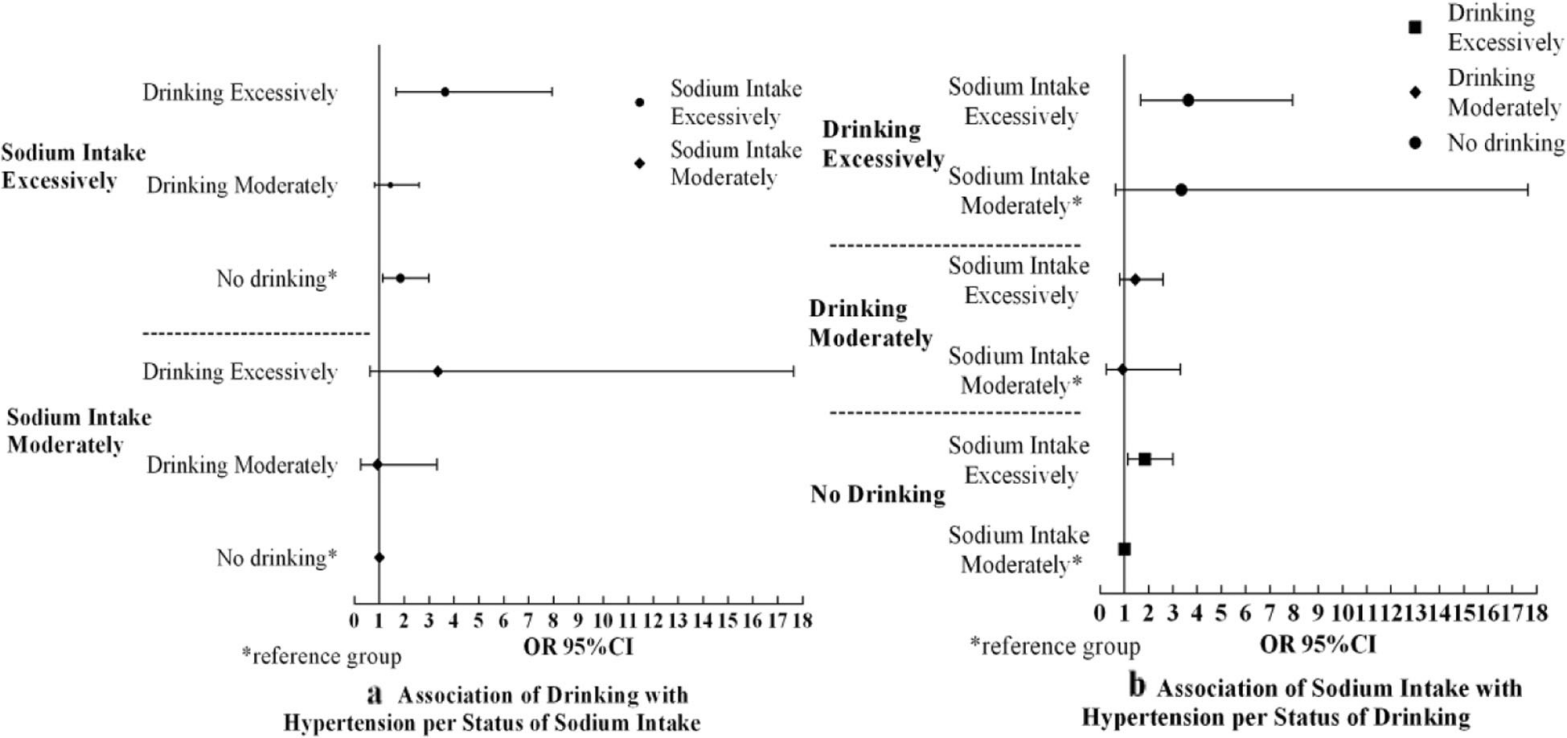
Effects of drinking and sodium intake on hypertension at different levels of these risk factors

Figure 2 shows the simple effect of drinking and sodium intake on hypertension at different levels. At the same level of sodium intake, excessive drinkers had a higher risk of hypertension than non-drinkers (Fig. 2a). Likewise, at the same level of drinking, the group with excessive sodium intake had a significantly higher risk of hypertension than those with a moderate sodium intake (*p* < 0.05) (Fig. 2b).

## Discussion

In this cross-sectional study, the standardized prevalence of hypertension was 62.33% among people aged ≥55 years in Inner Mongolia. The previously study reported that the hypertension prevalence was 54.6% in 31 other provinces in China [28]. Previous work has shown that the risk of hypertension increased with age, and those aged 55–60 years are the most likely to have hypertension [7]. Moreover, with increasing age, people are more susceptible to chronic depression and declines in body function caused by changes in metabolic status, which may influence the intensity of physical activity after age 55 [29]. Because of the characteristics of residents of Inner Mongolia who are aged 55 years and older, interventions for the prevention of hypertension should place significantly more emphasis on this population. Both excessive sodium intake and excessive drinking were independently associated with hypertension. Moreover, the combined effect of excessive sodium intake and excessive drinking was associated with the highest risk of hypertension, which suggests that preventive interventions including both sodium reduction and alcohol consumption control are very important.

The present study showed that sodium intake and drinking were both independently related to the risk of hypertension among a representative sample of Inner Mongolia residents. Compared with heavy alcohol consumption, excessive sodium intake was more closely related to hypertension, which was in accordance with other studies [30]. Our study also confirmed that excessive sodium intake is independently associated with the risk of hypertension. Another study reported that a reduction in adults’ sodium intake decreased systolic blood pressure by 3.39 mmHg, and this change was significant [31]. The effect of decreasing blood pressure by reducing sodium intake was more pronounced in older adults than in younger subjects. Interestingly, a 12-month follow-up study found that participants older than 55 years who used a salt substitute containing 65% sodium chloride had lower blood pressure than those who consumed normal salt (100% sodium chloride) [32].

Compared with being a non-drinker, being an excessive drinker was significantly independently associated with an increased risk of hypertension in this study. As was also shown in a previous study, regular alcohol consumption was associated with double the odds of hypertension compared with being a non-drinker [33]. The Atherosclerosis Risk in Communities cohort study revealed that the risk of hypertension was higher in people who drank more than 210 g of alcohol per week compared with non-drinkers [34]. However, we did not find a relationship between moderate drinking and hypertension. Even in the multivariate interaction analysis, the effect of moderate drinking on hypertension risk was relatively small. Currently, the impact of light/moderate alcohol consumption on blood pressure is a controversial question. Accordingly, Jaubert and colleagues evaluated the relationship between alcohol consumption and 24-h ambulatory blood pressure in a community-based older adult cohort. After adjusting for relevant covariates, they found that blood pressure was significantly higher in moderate-to-heavy drinkers than in the reference group [35]. However, the Global Burden of Disease 2016 Alcohol Collaborators found that consuming zero standard drinks daily was associated with the lowest risks in all aspects of health [11]; in other words, not drinking is the healthiest lifestyle. This suggests that further study is required to explore the effect of moderate alcohol consumption on hypertension.

We also found a significant multiplicative interaction effect of sodium intake and drinking on the risk of hypertension. Individuals who consumed both sodium and alcohol excessively had the highest risk of hypertension, compared with those with moderate sodium intake and no alcohol consumption. Similar to our findings, consuming excessive alcohol and high levels of salted fermented seafood has been found to be associated with significantly elevated rates of pre-hypertension and hypertension in Korean adults [36]. Participants consuming high levels of alcohol were 3.05 times more likely to be hypertensive than those with low consumption patterns. However, this previous study examined dietary patterns generated using factor analysis and the drinking dietary pattern of consuming alcohol and salted fermented seafood [36].. A similar study conducted in China found that drinking and dietary patterns focusing on the amount of alcohol and condiments including salt and oil consumed were associated with an increased risk of hypertension [37]. Alcohol intake was also found to cause significant increases in blood pressure and sodium balance in Japanese men with hypertension in a study by Yuhei and colleagues, which suggests that sodium and alcohol intake may interact with each other; however, this previous study did not clarify the specific mechanism of the interaction [38]. Salt-sensitive hypertension is common in older adults; in addition, long-term drinking can accelerate the development of hypertension by damaging the renin-angiotensin system and impairing endothelial function, resulting in decreased salt sensitivity. Gennaro and colleagues studied how blood pressure responded to dietary sodium disposal among alcoholics; interestingly, they found that the sodium sensitivity index, which measures the degree of salt sensitivity, was significantly higher in alcoholics, and that blood pressure was significantly elevated [39]. Another study showed that drinking excessively led to chronic sodium retention and increased intracellular sodium ion concentration, resulting in hypertension [40].

Consistent with our findings, previous studies have reported significant associations between excessive drinking and blood pressure level at the same level of sodium intake. We also found that, at the same level of drinking, excessive sodium intake was associated with an increased risk of hypertension across all alcohol consumption groups. This suggests that drinking is an influential factor that increases the association of sodium intake with the risk of hypertension.

Inner Mongolia is a multi-ethnic region in the most northern part of China, with large internal variations in geography and climate. Because the annual cold season is longer in this region than in other areas of China, the Inner Mongolia residents’ diet is characterized by lower intake of fresh fruit and vegetables and greater intake of foods containing oil and salt, and excessive drinking is a prevalent habit [21]. Wine is an essential beverage for social gatherings in Inner Mongolia, and higher alcohol consumption tends to increase energy intake because residents are accustomed to eating pickled food with high salt content when they drink. Our findings indicate that the consistent presence of both excessive sodium intake and heavy drinking greatly increases the risk of hypertension.

### Limitations

This study had several limitations. As is the case in all cross-sectional studies, the difficulty of the accurate evaluation of alcohol consumption and the possibility underreporting or over-reporting should be considered. It is likely that any classification or measurement error occurred at random, which may have attenuated the observed findings, leading to an underestimate of effects. Misclassification, particularly for the reporting of alcohol consumption, may have led to an underestimation of the effects of drinking on hypertension risk. Additionally, because this was a cross-sectional study, the causal associations of sodium intake and drinking with the risk of hypertension should be further examined in large cohort studies. Finally, the study sample was small. However, we endeavoured to minimise distortion and generalisation of the data by using statistical tests and sampling methods. Addition research with larger numbers of subjects is required to verify our findings.

## Conclusions

The findings of this study indicate that excessive sodium intake and excessive drinking are highly prevalent dietary habits among older adults in Inner Mongolia. The age-standardized rate of hypertension was 62.33% among the study participants, who were aged 55 years and above. Both sodium intake and alcohol consumption were independently associated with hypertension. More importantly, sodium intake was found to interact with drinking in a synergistic association with the risk of hypertension among those aged 55 years and above. Primary health care workers should be more inclined to provide interventions to reduce the risk of hypertension for older adults who have unhealthy eating habits and consume both sodium and alcohol excessively.

## Data Availability

The questionnaire and data are available in machine-readable format upon request from the corresponding author upon reasonable request, but they are not available online.

## Abbreviations

DALYs: Disability-adjusted life years
BMI: Body mass index
OR: Odds ratio
CI: Confidence interval
MET: Metabolic equivalent
